# Prognostic Immune-Related Genes of Patients With Ewing’s Sarcoma

**DOI:** 10.3389/fgene.2021.669549

**Published:** 2021-05-28

**Authors:** Yangfan Zhou, Bin Xu, Shusheng Wu, Yulian Liu

**Affiliations:** ^1^The First Affiliated Hospital of Anhui Medical University, Hefei, China; ^2^The First Affiliated Hospital of USTC, Hefei, China

**Keywords:** gene expression, immunology, ewing’s sarcoma, gene, immune microenvironment, prognosis

## Abstract

Ewing’s sarcoma (ES) is an extremely aggressive malignant bone tumor with a high incidence among children and adolescents. The immune microenvironment plays an important role in ES development. The aim of the current study was to investigate the immune microenvironment in ES patients to identify immune-related gene signatures. Single-sample gene set enrichment analysis (ssGSEA) was used to cluster the RNA sequences of 117 ES patients, and their immune cell infiltration data were downloaded and evaluated based on the Gene Expression Omnibus (GEO) database. High, medium, and low immune cell infiltration clusters were identified. Based on the comparison of clusters with high and low immune cell infiltration, normal skeletal muscle cells, and ES, we identified 198 common differentially expressed genes. GO and KEGG enrichment analyses indicated the underlying immune mechanism in ES. Cox and LASSO regression analyses were conducted to select immune-related prognostic genes. An external dataset from the International Cancer Genome Consortium (ICGC) was used to validate our results. Ten immune-related, independent prognostic genes (*FMO2, GLCE, GPR64, IGFBP4, LOXHD1, PBK, SNAI2, SPP1, TAPT1-AS1*, and *ZIC2)* were selected for analysis. These 10 immune-related genes signature were determined to exhibit independent prognostic significance for ES. The results of this study provide an approach for predicting the prognosis and survival of ES patients, and the elucidated genes may be a promising target for immunotherapy.

## Introduction

Ewing’s sarcoma (ES) is an extremely aggressive malignant bone tumor with a high incidence among children and adolescents (Subbiah et al., 2009; Gupta et al., 2010). Primary bone tumors account for 5% of all childhood and adolescent cancers. ES is the second most commonly reported primary bone tumor (Balamuth and Womer, 2010). In previous studies (Jiménez-Morales et al., 2009), immune-related genes and immune cells were found to be closely related to the occurrence and development of autoimmune diseases. For example, the TNF-β-308a allele is a common genetic risk factor for the development of childhood immune and/or inflammatory diseases (Jiménez-Morales et al., 2009). Synovitis caused by rheumatoid arthritis is also closely associated with the infiltration of various immune cells (Weyand and Goronzy, 2021).

Currently, immuno-oncology has attracted considerable attention owing to its role documented in various cancers. Tumor tissue contains a substantial number of immune cells, such as macrophages, T cells, and NK cells, which infiltrate the tumor microenvironment. These cells secrete a variety of factors that affect the microenvironment of the tumor, a phenomenon known as immune infiltration, which regulates tumor behavior and exhibits potential prognostic value (Singh et al., 2017). Immunotherapy is a new treatment method that has achieved appreciable results in breast cancer, hepatocellular carcinoma, and other cancers (Guerra et al., 2017; Liu et al., 2018). Immune cell infiltration also plays an important role in the occurrence and development of ES. For example, *EZH2* inhibitors can sensitize ES cells to effective cytolysis through the action of GD2-specific *CAR* gene-modified T cells. Therefore, a strategy involving the adoptive transfer of GD2-redirected T cells to ES demonstrates efficacy (Kailayangiri et al., 2019). Berghuis et al. (2011) found that inflammatory chemokines (CXCR3, CCR5, CXCL9, CXCL10, and CCL5) could recruit CD8+ T cells for immune infiltration in ES. They recognized that the development of an inflammatory microenvironment might increase the efficacy of the natural immune response to ES (Berghuis et al., 2011). However, there are few studies available on immunotherapy targets, prognostic biomarkers, and immunotherapy programs whose approaches have been applied to ES.

Therefore, a comprehensive analysis of the prognosis of immune infiltration in ES patients is warranted to provide insights into new targets and approaches for the treatment of ES. The objective of the current study was to investigate the immune microenvironment of ES patients and to identify immune-related gene signatures by using ssGSEA (Subramanian et al., 2005) to divide ES patients into high, medium, and low immune cell infiltration groups. Then, we identified a 10 immune-related prognosis genes signature correlated with the prognosis in differentially expressed genes (DEGs) in both ES group and high immune cell infiltration cluster by Cox regression and least absolute shrinkage and selection operator (LASSO) regression. Finally, we used internal and external dataset to evaluate the accuracy of the prognosis of immune-related genes. Additionally, immune-related gene signatures improve the prognostic predictive ability of ES patients and help to elucidate the underlying mechanism involved in the disease.

## Materials and Methods

### ES Data Download

**FIGURE 1:**
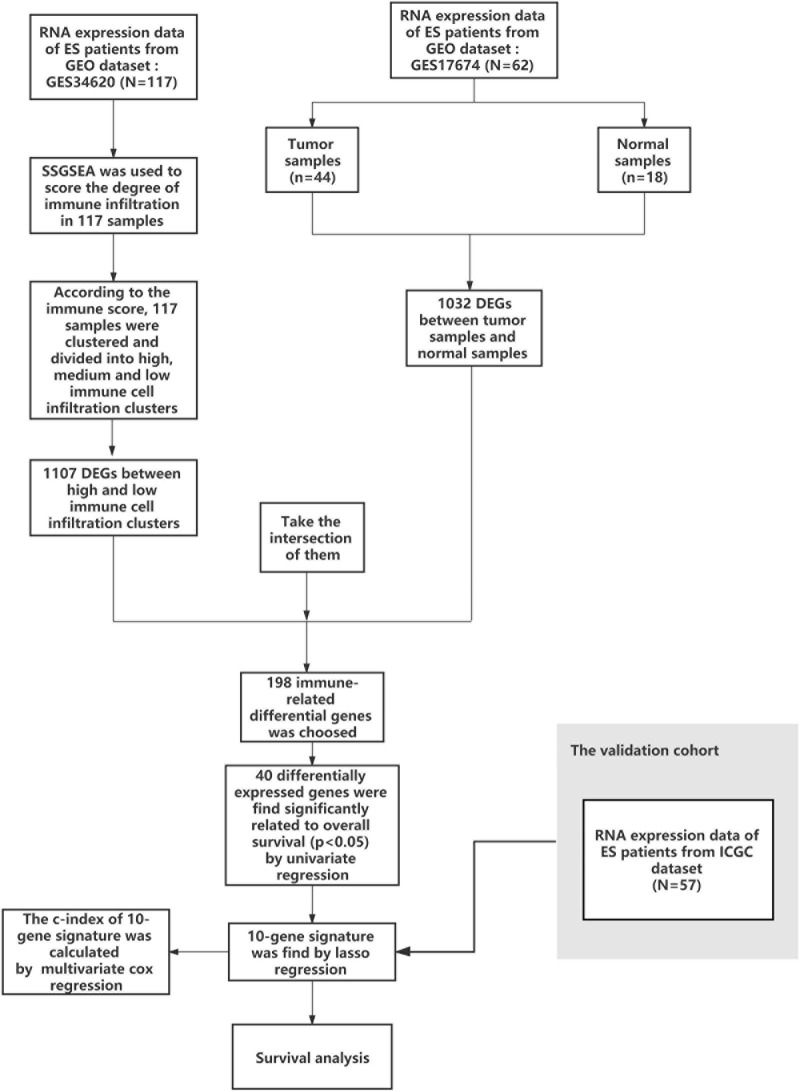
Flow chart of data collection and analysis.

A working flow chart is depicted in Figure 1. In this study, we downloaded the ES dataset from the NCBI Gene Expression Omnibus (GEO) database^1^. The accession numbers were GSE17674 and GSE34620 (Savola et al., 2011; Postel-Vinay et al., 2012), and the data platform numbers were both GPL570. GSE17674 contained RNA-sequencing data and patient survival information of 44 ES samples, and RNA-sequencing data of 18 normal human skeletal muscle samples. GSE34620 contained RNA-sequencing data of 117 ES samples. Additionally, we used data on 57 ES samples from the ICGC^2^ for external validation of prognostic genes (Alexandrov et al., 2020).

### Data Clustering

The data on gene set of 28 immune-related cells were obtained from the literature (Jia et al., 2018). We used the Gene Set Variation Analysis (GSVA) R package (Hänzelmann et al., 2013) to investigate the degrees of infiltration of different immune cell types in the ES gene expression profile of GSE34620. Data were subjected to an unsupervised hierarchical clustering algorithm using the function “hclust.” The function named “ColorDendrogram” of R package “sparcl” was used to draw a clustering tree (cut off = 1.0), and aided the categorization of ES samples into high, medium, and low immune cell infiltration clusters.

### Validation of the Effectuality of Immune Clustering

Yoshihara et al. had presented a new algorithm that takes advantage of the unique properties of the transcriptional profiles of cancer samples to infer tumor cellularity as well as the different infiltrating normal cells, called ESTIMATE (Estimation of STromal and Immune cells in MAlignant Tumor tissues using Expression data). The ESTIMATE algorithm was utilized based on the gene expression signatures to infer the fraction of stromal and immune cells in tumor samples by calculating stromal and immune scores to predict the level of infiltrating cells. stromal and immune scores to predict the level of infiltrating stromal and immune cells and these form the basis for the ESTIMATE score to infer tumor purity in tumor tissue. This indicated tumor purity, which was inversely proportional to immune scores, stromal scores and estimate score (Yoshihara et al., 2013). First, the R package “ESTIMATE” was used to elucidate the tumor purity, estimated score, immune score, and stromal score. Then the R package “ggpubr” (Whitehead et al., 2019) was used to generate a violin plot of the data on tumor purity, evaluation score, immune score, and the interstitial score of the high, middle, and low immune cell infiltration clusters to verify effectuality of the immune infiltration clusters and to illustrate a clustered heatmap. Additionally, we verified the differences between the three immune infiltration groups through the expression of the members of HLA family members and PD-L1 (CD274), and we performed *p*-value correction for multiple testing by using the Holm–Bonferroni method.

### GSEA Enrichment Analysis

The R package “clusterprofiler” (Yu et al., 2012) was used to perform Gene Ontology (GO) and Kyoto Encyclopedia of Genes and Genomes (KEGG) pathway enrichment analysis of the high and low immune cell infiltration clusters of the GEO database. The R package “enrichplot” was used to generate annotated bubble charts. *p* < 0.05 was considered statistically significant.

### Distinction of Immune-Related Genes in ES

Based on the above-mentioned clustering, the mRNA-seq expression data of GSE34620 were divided into the following three types: high, medium, and low immune cell infiltration. We used the R package “limma” (Ritchie et al., 2015) to obtain data on the DEGs of high and low immune cell infiltration in the database. We used the Benjamini-Hochberg method to obtain the false discovery rate (FDR) (Hochberg and Benjamini, 1990) (| logFC| > 2 and adj.*p*-value < 0.05, where FC is the fold change). Additionally, data on the DEGs (| logFC| > 2 and adj.*p*-value < 0.05) between normal skeletal muscle and ES samples in GSE17674 were analyzed. Finally, we plotted Venn diagrams to visualize the data on immune-related differential genes in GSE34620 and the differential genes in GSE17674.

### Distinction and Confirmation of Immune-Related Gene Prognostic Signatures for ES

First, univariate Cox analysis of overall survival (OS) was performed to screen immune-related genes with prognostic values in GES17674. The LASSO algorithm was used for variable selection and shrinkage using the R package “glmnet” (Friedman et al., 2010). A 1,000-round cross-validation for tuning the parameter selection was performed to minimize the risk of overfitting. Then, multivariate Cox regression analysis was performed for the data on the genes obtained from LASSO regression analysis, and a forest map was constructed using the R package “survminer”. Finally, immune-related gene prognostic signature of ES were constructed. The formula used was as follows:

R⁢i⁢s⁢k⁢⁢s⁢c⁢o⁢r⁢e=∑coefficient⁢(genei)⁢×expression⁢(genei)

Using “Survminer” R package, ES was divided into high-risk and low-risk groups according to the median of the risk score. The time-dependent receiver-operating characteristic (ROC) and Kaplan-Meier (K-M) curves were used to assess the clinical prognostic capacity of the risk score using the R packages “timeROC” and “survival”. Additionally, we used the verification set from the ICGC to externally verify the feasibility of this risk level.

### Construction and Verification of Nomogram

Based on the risk level assessed by performing multivariate Cox regression and by using the patient’s clinical information, we used the R packages “rms” and “survival” to construct a new prognostic nomogram. The concordance index (C-index) value was used to evaluate the predictive performance of the nomogram. The value of the C-index ranged from 0.5–1.0, with 1 indicating the best prediction of the model. A C-index value over 0.7, which implies a relatively accurate prediction, and calibration curves are often used to assess the accuracy of the nomogram, and the distance between pairs and the 45-degree line is a measure of the absolute error of the nomogram prediction (Iasonos et al., 2008). Therefore, the C-index value and calibration curves were used to validate the model. Additionally, we externally verified the constructed nomogram using the ICGC verification set.

## Results

### Construction and Validation of ES Clustering

**FIGURE 2:**
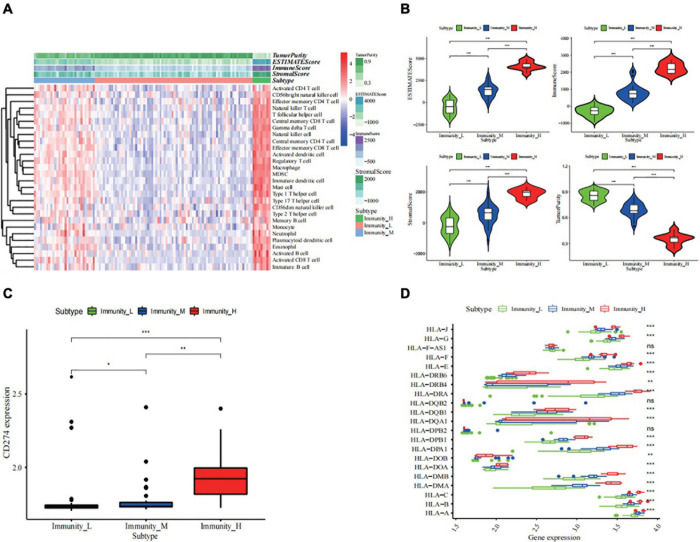
Construction and validation of ES clustering. **(A)** The enrichment levels of 28 immune-related cells in the high immune cell infiltration group (Immunity_H), middle immune cell infiltration group (Immunity_M), and the low immune cell infiltration group (Immunity_L). The Tumor Purity, ESTIMATE Score, Immune Score and Stromal Score of every patient gene were showed combine with the clustering information. **(B)** The violin plot showed the difference in ESTIMATE Score, Immune Score, Stromal Score, and Tumor Purity between three clusters. The statistical method is wilcoxon test **(C,D)** The kruskal-wallis test showed the expression of most HLAs and the wilcoxon test showed PD-L1 (CD274) was a significant difference in high- (red), middle- (blue), and low- (green) immune cell infiltration cluster. na adj.*p* > 0.05, * adj.*p* < 0.05, ** adj.*p* < 0.01, *** adj.*p* < 0.001. ns, no statistically significant., *p*-value correction for multiple testing.

Based on the gene set containing 28 immune cells, 117 ES samples from GSE34620 were enriched and scored using ssGSEA. Hence, the samples were grouped into three clusters, namely high, middle, and low levels of immune infiltration (Supplementary Figure 1 and Figure 2), of which there were nine, thirty, and seventy-eight cases, respectively (Figure 2A). The results showed that the stromal, immune, and estimated scores of the high immune cell infiltration group were higher than those of the other two clusters, while the tumor purity showed the opposite trend (Figure 2B and Supplementary Table 1). Additionally, we described the expression levels of most members of the HLA family and PD-L1 (CD274) in the high, middle, and low clusters using box plots (*p* < 0.05, Figures 2C,D).

### GSEA Enrichment Analysis

**FIGURE 3:**
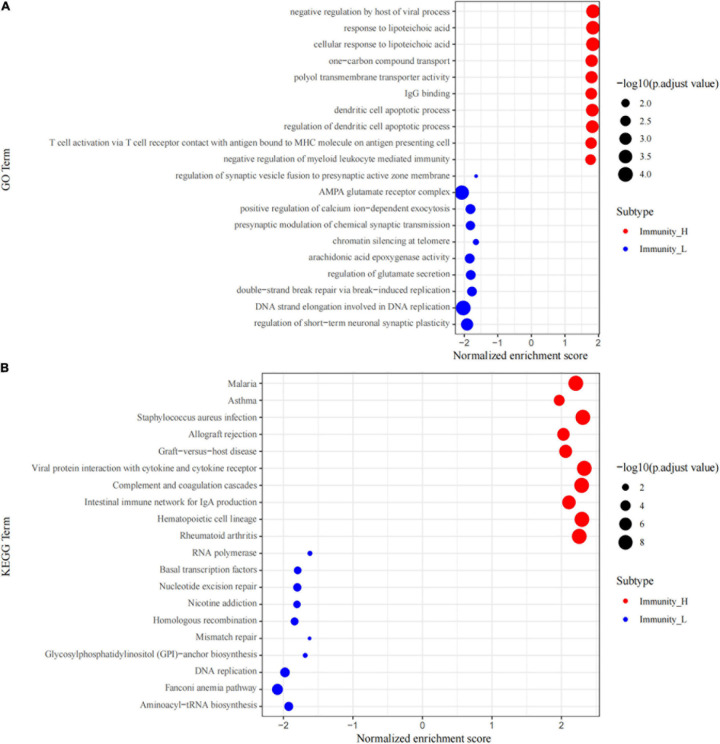
GSEA enrichment analysis. **(A)** The top 10 results of GO analysis in Immunity_H (red) and Immunity_L (green). **(B)** The results of KEGG analysis in Immunity_H (red) and Immunity_L (green).

The GO and KEGG pathway analyses of genes in the high and low immune cell infiltration clusters in GSE34620 showed that several immune-related molecular functions were enriched in the cohort. These inclued negative regulation by the host of viral processes, IgG binding, T cell activation via T cell-receptor contact with antigen bound to MHC molecules on antigen presenting cells, and negative regulation of myeloid leukocyte-mediated immunity (Figure 3A). KEGG analysis results showed that these genes were related to pathways, such as viral protein interaction with cytokines and its receptors, intestinal immune network for IgA production, graft-versus-host disease, and Staphylococcus aureus infection (Figure 3B).

### Identification of Immune-Related Differentially Expressed Genes Between the ES and Normal Groups

**FIGURE 4:**
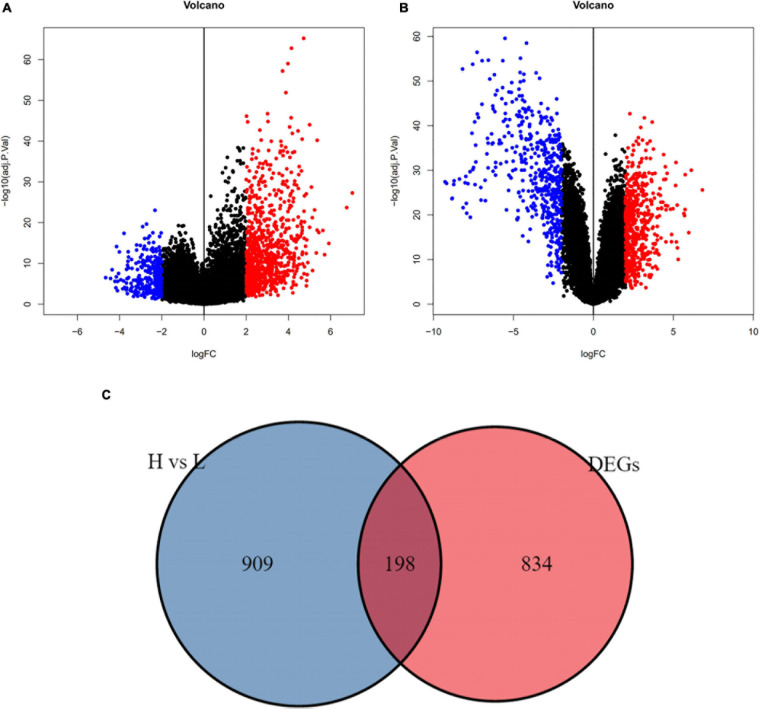
Immune – related differential genes were defined. **(A)** The volcano plot showed different genes between high and low immune cell infiltration cluster (adj.*p*-value < 0.05). **(B)** The volcano plot showed different genes between ES and normal skeletal muscle cells (adj.*p*-value < 0.05). **(C)** Using draw Venn diagram to pick up the intersection, 198 differentially expressed genes were obtained.

In GSE34620, we obtained 1107 DEGs between the high and low immune infiltration groups (| logFC| > 2 and adj. *p*-value < 0.05, Figure 4A). Additionally, we identified the DEGs between ES and normal skeletal muscle in the GSE17674 cohort. A total of 1032 DEGs were obtained (| logFC| > 2 and adj.*p*-value < 0.05; Figure 4B). Venn diagrams visualizes 198 immune-related DEGs (Figure 4C).

### Screening and Identification of 10 Immune-Related Prognostic Genes

**FIGURE 5:**
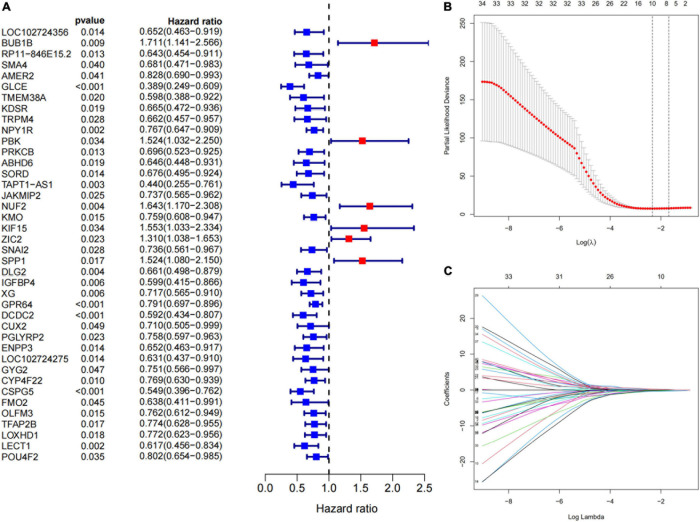
Construction of immune-related genes prognostic signature. **(A)** The *p*-value and HR of selected genes in univariable Cox regression analysis (*p* < 0.05). **(B)** The LASSO regression identified 10 genes associated with prognosis. The partial likelihood deviance plot presented the minimum number corresponds to the covariates used for LASSO Cox analysis. **(C)** A coefficient profile plot was generated against the log (lambda) sequence. Selection of the optimal parameter (lambda) in the LASSO model for GEO cohort.

We selected 44 ES patients with complete clinical data from GSM17674 for further analysis. A univariate Cox regression analysis revealed that 40 of the 198 DEGs related to immunity were significantly associated with OS (*p* < 0.05, Figure 5A). Then LASSO regression analysis aided in the identification of the *FMO2, GLCE, GPR64, IGFBP4, LOXHD1, PBK, SNAI2, SPP1, TAPT1-AS1*, and *ZIC2 10* genes (Figures 5B,C). Finally, according to the multivariate Cox regression, 10 prognostic signatures of ES were constructed. We scored the risk according to the risk coefficients of these 10 genes as well as their expression. The formula is:

=FMO2×(-0.869033333)+GLCE×(-0.445266086)

+GPR64×(-0.262532651)+IGFBP4×(-0.506728078)

+LOXHD1×(-0.217842143)+PBK×(0.005800429)

+SNAI2×(0.054535428)+SPP1×(0.175590092)

+TAPT1-AS1×(-0.274747367)+ZIC2×(0.704860559).

**FIGURE 6:**
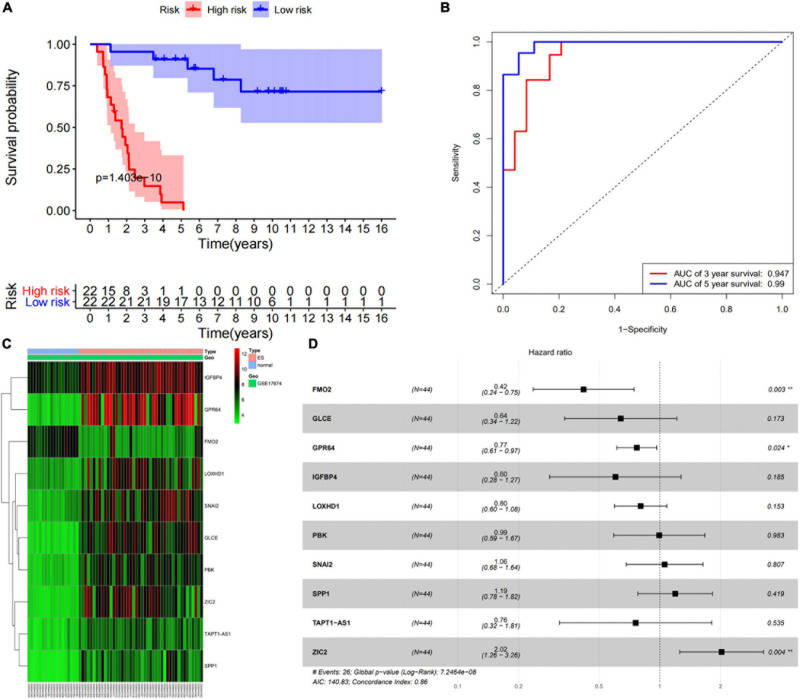
Prognostic value of 10 immune-related gene signatures. **(A)** The survival curve show that low risk level has better prognosis in training set (GES17674). **(B)** The ROC curve for 3-and 5-overall survival of ES patients in training set (GES17674). **(C)** Heatmap showed the expression level of 10 genes in the normal group and ES group. **(D)** The results of multivariate Cox regression of 10 genes.

**FIGURE 7:**
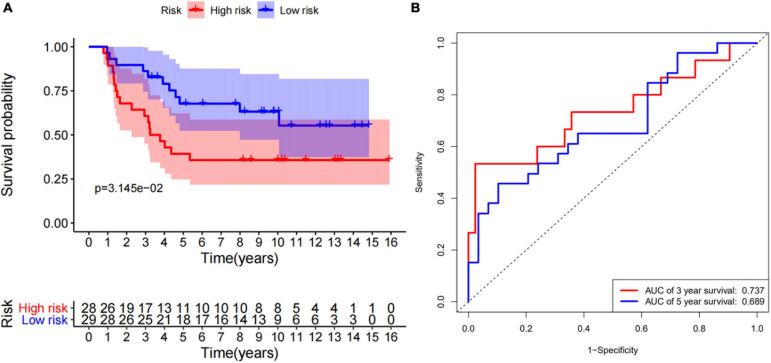
10 immune-related genes signature is validated by ICGC cohort. **(A)** The survival curve show that low risk level has better prognosis in verfication set (ICGC). **(B)** The ROC curve for 3-and 5-overall survival of ES patients in verfication set (ICGC).

According to the median of the risk scores, GSM17674 was divided into high-risk and low-risk groups. The K-M curve showed that the survival rate of the low-risk group was significantly higher. In the high-risk score group (*p* < 0.001, Figure 6A), the risk score exhibited an effective progonstic value for prognosis. Additionally, the time-dependent ROC curves were used to evaluate the nine immune-related gene signals in predicting the total 3–5-year survival of ES patients. The area under the ROC value (AUC) value for 3 and 5 years was 0.99 and 0.947, respectively (Figure 6B), indicating that the 10 immune-related genes showed appreciable ability to predict OS. Based on the differential expression of the genes in the normal and ES groups, a heatmap was illustrated in which only *FMO2* expression was upregulated (Figure 6C). Additionally, all gene signatures were analyzed by multivariate Cox regression, among which *FMO2*, *GPR64*, and *ZIC2* were statistically significant (*p* < 0.05), and the C-index of the model was 0.86 (Figure 6D). Moreover, patients in the ICGC cohort were also divided into high-risk or low-risk groups according to the median calculated using the same formula as the GEO cohort. Survival analysis showed that patients in the low-risk group had a higher survival rate (Figure 7A). The AUCs of 3- and 5-year OS were 0.737 and 0.689, respectively (Figure 7B).

### Construction and Verification of Nomogram

**FIGURE 8:**
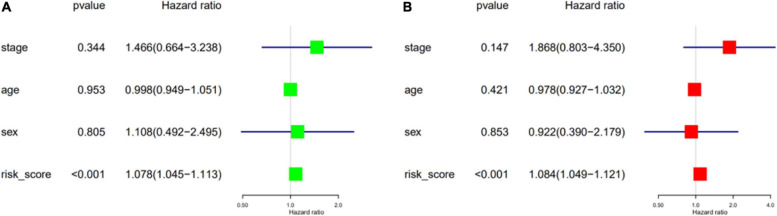
Validation of independent prognostic factors. The univariate **(A)** and multivariate **(B)** Cox regression analysis of risk score, age, gender, and stage.

**FIGURE 9:**
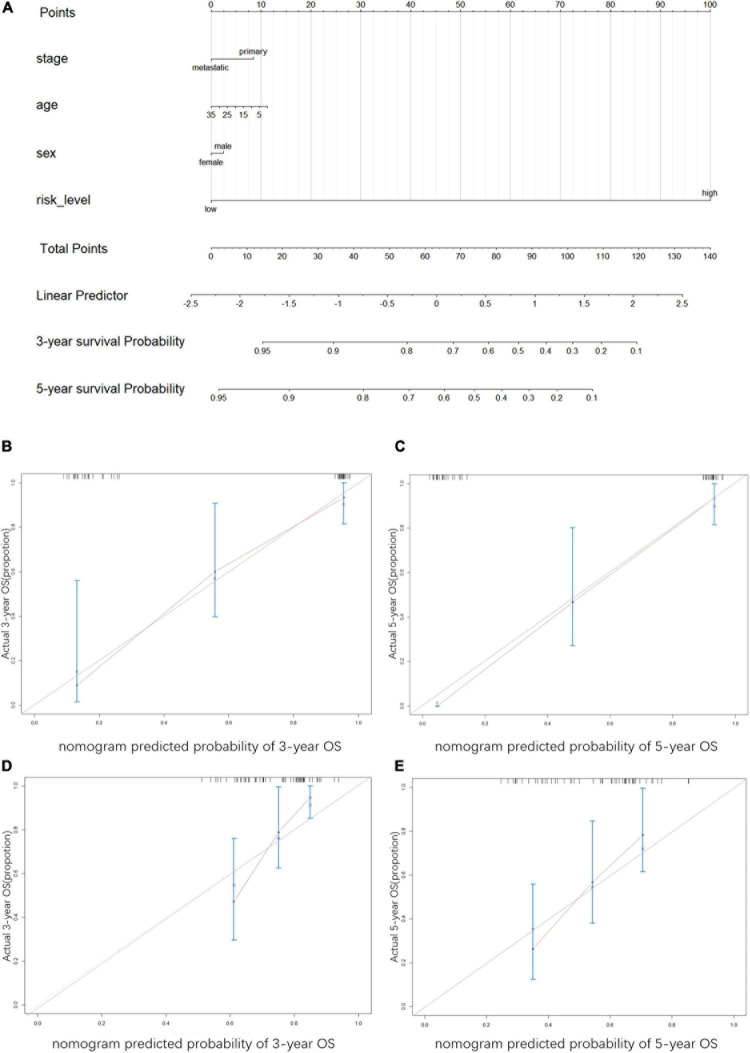
Construction and verification of nomogram **(A)** Nomogram was constructed to predict the survival time. The risk level, age, gender, and metastasis status were used as variables. 1. Names of the variables in the prediction model: for example, age, gender, stage, and risk level. Each variable corresponds to a line segment marked with a scale, representing the range of values that can be taken for that variable, while the length of the line segment reflects the size of the contribution of that factor to the outcome event. 2. Scores, including individual scores; “Points” in the figure, which represent the individual scores corresponding to each variable at different values; and total scores. “Total Points”, which represents the total scores of the individual scores corresponding to all variables taken together. 3. Predicted probability: the 3- and 5-year survival probability in the figure. The graphs show the calibration plots for internal validation of **(B)** actual 3-year OS and **(C)** 5-year OS; and for extral verfication of **(D)** actual 3-year OS and **(E)** actual 5-year OS. The vertical coordinate represents the actual overall survival while the horizontal coordinate represents the predicted overall survival, the red line is the fitted line, which indicates the actual value corresponding to the predicted value, small ticks at the top of each plot representative ES patients, and the three vertical lines connected by the red lines represents the estimated fluctuation of each point.

We performed univariate and multivariate Cox regression analyses to ascertain the independence of the risk score as a prognostic factor for other features, such as sex, age, and metastasis status (Figures 8A,B). Based on the risk level and the patient’s clinical information, a new prognostic nomogram was constructed (Figure 9A), in which the internal verification in the GEO cohort C-index was 0.814 and the external verification in the ICGC cohort was 0.66. Additionally, the internal and external 3- and 5-year calibration curves reflected the good predictive ability of the model (Figures 9B–E).

## Discussion

In previous studies conducted on other sarcomas, immune infiltration has been reported to be closely related to the patient prognosis (Chen et al., 2020; Xiao et al., 2020). Immunotherapy can activate the patient’s own immune system to eliminate tumor cells and has become the most anticipated tumor treatment method. In this study, we evaluated patients with different degrees of immune infiltration, and immune-related prognostic genes were selected to provide new directions for future ES treatment.

The majority of the immune-related prognostic genes are related to the occurrence and development of different types of cancer. In neck squamous cell carcinoma, miR-205-5p may affect the occurrence and development of neck squamous cell carcinoma by acting on the target gene *FMO2* (Zhou et al., 2020). As a potential tumor suppressor gene, *GLCE* participates in the occurrence and development of breast and lung cancer, mainly through the inhibition of tumor angiogenesis and invasion/metastasis pathways. However, the increased expression of *GLCE* is associated with advanced pathophysiology of prostate tumors, which indicates that the role of *GLCE* in different cancers is diverse (Rosenberg et al., 2014). According to our research, the upregulation of *GLCE* is related to the poor prognosis of ES, which suggests that the mechanism of action of this gene in ES is similar to that observed in prostate tumors. Previous studies have shown that *GPR64* promotes the invasion and metastasis of Ewing’s sarcoma through PGF and MMP1 (Richter et al., 2013). However, *GPR64* exerts an important tumor suppressor effect in endometrial cancer (Richter et al., 2013), suggesting that this gene plays different roles in various tumors. In liver and breast cancer, *IGFBP4* inhibits growth and invasion, and its overexpression is often associated with a better prognosis (Lee et al., 2018; Wang et al., 2019). *PBK* knockdown exerted no effect on the proliferation of gastric cancer cells, although it inhibited cell migration and invasion. However, overexpression of *PBK* is associated with a worse prognosis (Kwon et al., 2016). The transcription factor Snai2 encoded by *SNAI2* is an evolutionarily conserved C2H2 zinc finger protein that co-ordinates biological processes critical for tissue development and tumorigenesis (Zhou et al., 2019). Studies have found that *SOX13* can promote colorectal cancer metastasis by activating *SNAI2* and *c-MET* (Du et al., 2020). *SNAI2* reprograms stromal fibroblasts, and this contributes to tumor proliferation and ovarian cancer progression (Yang et al., 2017). *SPP1* expression is closely related to immunity in lung cancer, ediates the polarization of macrophages, and promotes the immune escape of lung adenocarcinoma via upregulation of *PD-L1*, leading to the metastasis of lung cancer cells (Zhang et al., 2017). Additionally, SPP1 expression is correlated with the proliferation of ES cells as it is an immune-related hub gene (Ren et al., 2021). This is consistent with our findings, suggesting that *SPP1* may be a potential therapeutic target for ES. *ZIC2* can be utilized as a useful prognostic indicator in breast cancer and exerts a tumor suppressor effect by regulating *STAT3* (Liu et al., 2020). Additionally, *ZIC2* is used to inhibit breast cancer cell proliferation, migration, and invasion as it is a target gene of miR-1284 (Zhang et al., 2019). However, there are few studies available on *LOXHD1* and *TAPT1-AS1*; hence, these genes should be the subject of further investigation to identify new therapeutic targets for ES.

Gene signatures were constructed for the 10 immune-related genes with prognostic value. Based on the risk score, patients in the high-risk score group had a worse prognosis. Univariate and multivariate Cox regression analyses were performed with the clinical factors of age, sex, and tumor development stage. The risk score could be considered an independent prognostic factor of ES (*p* < 0.01), and the internal and external survival curves and AUC values verified the good predictive ability observed therein.

Finally, a novel ES prognostic nomogram was constructed according to the risk score level, age, gender, and tumor development stage, and the calibration curve and C-index values (validated internally and externally) showed the model’s good predictive ability.

Although these findings provide valuable information for a predictive prognosis in ES, there are certain limitations of the present study. First, due to the rarity of ES, the GEO data in our training set are relatively limited, although we have performed the external verification of the ICGC dataset to compensate for this shortcoming. Second, additional external data and experiments are warranted to verify our results as only public datasets have been used. Overall, we elucidated the immune-related prognostic genes of ES. Our research provides new aspects for future research on ES. The 10 genes identified in this study may serve as new therapeutic targets for ES.

## Conclusion

These 10 immune-related gene signatures demonstrated independent prognostic significance for ES. The results of this study provide an approach for predicting the prognosis and survival of ES patients and these genes serve as a promising target for immunotherapy.

## Data Availability Statement

The datasets presented in this study can be found in online repositories. The names of the repository/repositories and accession number(s) can be found in the article/Supplementary Material.

## Author Contributions

YZ: data processing and article writing. SW: provide data, data processing, and article writing. YL: article writing. BX: provide data. All authors contributed to the article and approved the submitted version.

## Conflict of Interest

The authors declare that the research was conducted in the absence of any commercial or financial relationships that could be construed as a potential conflict of interest.

## Footnotes

1 http://www.ncbi.nlm.nih.gov/geo

2 https://icgc.org

## References

[B1] Alexandrov L. B. Kim J. Haradhvala N. J. Huang M. N. Tian Ng A. W. Wu Y. (2020). The repertoire of mutational signatures in human cancer. *Nature* 578 94–101. 10.1038/s41586-020-1943-3 32025018 PMC7054213

[B2] Balamuth N. J. Womer R. B. (2010). Ewing’s sarcoma. *Lancet Oncol.* 11 184–192. 10.1016/s1470-2045(09)70286-4 20152770

[B3] Berghuis D. Santos S. J. Baelde H. J. Taminiau A. H. Egeler R. M. Schilham M. W. (2011). Pro-inflammatory chemokine-chemokine receptor interactions within the Ewing sarcoma microenvironment determine CD8(+) T-lymphocyte infiltration and affect tumour progression. *J. Pathol.* 223 347–357. 10.1002/path.2819 21171080

[B4] Chen H. Song Y. Deng C. Xu Y. Xu H. Zhu X. (2020). Comprehensive analysis of immune infiltration and gene expression for predicting survival in patients with sarcomas. *Aging (Albany N. Y.)* 13 2168–2183. 10.18632/aging.202229 33316779 PMC7880383

[B5] Du F. Li X. Feng W. Qiao C. Chen J. Jiang M. (2020). SOX13 promotes colorectal cancer metastasis by transactivating SNAI2 and c-MET. *Oncogene* 39 3522–3540. 10.1038/s41388-020-1233-4 32111984

[B6] Friedman J. Hastie T. Tibshirani R. (2010). Regularization paths for generalized linear models via coordinate descent. *J. Stat. Softw.* 33 1–22. 20808728 PMC2929880

[B7] Guerra A. D. Yeung O. W. H. Qi X. Kao W. J. Man K. (2017). The anti-tumor effects of M1 macrophage-loaded poly (ethylene glycol) and gelatin-based hydrogels on hepatocellular carcinoma. *Theranostics* 7 3732–3744. 10.7150/thno.20251 29109772 PMC5667344

[B8] Gupta A. A. Pappo A. Saunders N. Hopyan S. Ferguson P. Wunder J. (2010). Clinical outcome of children and adults with localized Ewing sarcoma: impact of chemotherapy dose and timing of local therapy. *Cancer* 116 3189–3194. 10.1002/cncr.25144 20564643

[B9] Hänzelmann S. Castelo R. Guinney J. (2013). GSVA: gene set variation analysis for microarray and RNA-seq data. *BMC Bioinformatics* 14:7. 10.1186/1471-2105-14-7 23323831 PMC3618321

[B10] Hochberg Y. Benjamini Y. (1990). More powerful procedures for multiple significance testing. *Stat. Med.* 9 811–818. 10.1002/sim.4780090710 2218183

[B11] Iasonos A. Schrag D. Raj G. V. Panageas K. S. (2008). How to build and interpret a nomogram for cancer prognosis. *J. Clin. Oncol.* 26 1364–1370. 10.1200/jco.2007.12.9791 18323559

[B12] Jia Q. Wu W. Wang Y. Alexander P. B. Sun C. Gong Z. (2018). Local mutational diversity drives intratumoral immune heterogeneity in non-small cell lung cancer. *Nat. Commun.* 9:5361. 10.1038/s41467-018-07767-w 30560866 PMC6299138

[B13] Jiménez-Morales S. Velázquez-Cruz R. Ramírez-Bello J. Bonilla-González E. Romero-Hidalgo S. Escamilla-Guerrero G. (2009). Tumor necrosis factor-alpha is a common genetic risk factor for asthma, juvenile rheumatoid arthritis, and systemic lupus erythematosus in a Mexican pediatric population. *Hum. Immunol.* 70 251–256. 10.1016/j.humimm.2009.01.027 19480843

[B14] Kailayangiri S. Altvater B. Lesch S. Balbach S. Göttlich C. Kühnemundt J. (2019). EZH2 inhibition in ewing sarcoma upregulates G(D2) expression for targeting with gene-modified T Cells. *Mol. Ther.* 27 933–946. 10.1016/j.ymthe.2019.02.014 30879952 PMC6520468

[B15] Kwon C. H. Park H. J. Choi Y. R. Kim A. Kim H. W. Choi J. H. (2016). PSMB8 and PBK as potential gastric cancer subtype-specific biomarkers associated with prognosis. *Oncotarget* 7 21454–21468. 10.18632/oncotarget.7411 26894977 PMC5008298

[B16] Lee Y. Y. Mok M. T. Kang W. Yang W. Tang W. Wu F. (2018). Loss of tumor suppressor IGFBP4 drives epigenetic reprogramming in hepatic carcinogenesis. *Nucleic Acids Res.* 46 8832–8847. 10.1093/nar/gky589 29992318 PMC6158508

[B17] Liu Y. Qiao L. Zhang S. Wan G. Chen B. Zhou P. (2018). Dual pH-responsive multifunctional nanoparticles for targeted treatment of breast cancer by combining immunotherapy and chemotherapy. *Acta Biomater.* 66 310–324. 10.1016/j.actbio.2017.11.010 29129789

[B18] Liu Z. H. Chen M. L. Zhang Q. Zhang Y. An X. Luo Y. L. (2020). ZIC2 is downregulated and represses tumor growth via the regulation of STAT3 in breast cancer. *Int. J. Cancer* 147 505–518. 10.1002/ijc.32922 32064600

[B19] Postel-Vinay S. Véron A. S. Tirode F. Pierron G. Reynaud S. Kovar H. (2012). Common variants near TARDBP and EGR2 are associated with susceptibility to Ewing sarcoma. *Nat. Genet.* 44 323–327. 10.1038/ng.1085 22327514

[B20] Ren E. H. Deng Y. J. Yuan W. H. Wu Z. L. Zhang G. Z. Xie Q. Q. (2021). An immune-related gene signature for determining Ewing sarcoma prognosis based on machine learning. *J. Cancer Res. Clin. Oncol.* 147 153–165. 10.1007/s00432-020-03396-3 32968877 PMC11802167

[B21] Richter G. H. Fasan A. Hauer K. Grunewald T. G. Berns C. Rössler S. (2013). G-Protein coupled receptor 64 promotes invasiveness and metastasis in Ewing sarcomas through PGF and MMP1. *J. Pathol.* 230 70–81. 10.1002/path.4170 23338946

[B22] Ritchie M. E. Phipson B. Wu D. Hu Y. Law C. W. Shi W. (2015). limma powers differential expression analyses for RNA-sequencing and microarray studies. *Nucleic Acids Res.* 43:e47. 10.1093/nar/gkv007 25605792 PMC4402510

[B23] Rosenberg E. E. Prudnikova T. Y. Zabarovsky E. R. Kashuba V. I. Grigorieva E. V. (2014). D-glucuronyl C5-epimerase cell type specifically affects angiogenesis pathway in different prostate cancer cells. *Tumour Biol.* 35 3237–3245. 10.1007/s13277-013-1423-6 24264315

[B24] Savola S. Klami A. Myllykangas S. Manara C. Scotlandi K. Picci P. (2011). High expression of complement component 5 (C5) at tumor site associates with superior survival in Ewing’s sarcoma family of tumour patients. *ISRN Oncol.* 2011:168712. 10.5402/2011/168712 22084725 PMC3196920

[B25] Singh R. Mishra M. K. Aggarwal H. (2017). Inflammation. Immunity, and Cancer. *Mediators Inflamm.* 2017:6027305. 10.1155/2017/6027305 29234189 PMC5695028

[B26] Subbiah V. Anderson P. Lazar A. J. Burdett E. Raymond K. Ludwig J. A. (2009). Ewing’s sarcoma: standard and experimental treatment options. *Curr. Treat. Options Oncol.* 10 126–140. 10.1007/s11864-009-0104-6 19533369

[B27] Subramanian A. Tamayo P. Mootha V. K. Mukherjee S. Ebert B. L. Gillette M. A. (2005). Gene set enrichment analysis: a knowledge-based approach for interpreting genome-wide expression profiles. *Proc. Natl. Acad. Sci. U.S.A.* 102 15545–15550. 10.1073/pnas.0506580102 16199517 PMC1239896

[B28] Wang J. Luo X. X. Tang Y. L. Xu J. X. Zeng Z. G. (2019). The prognostic values of insulin-like growth factor binding protein in breast cancer. *Medicine (Baltimore)* 98:e15561. 10.1097/md.0000000000015561 31083221 PMC6531130

[B29] Weyand C. M. Goronzy J. J. (2021). The immunology of rheumatoid arthritis. *Nat. Immunol.* 22 10–18. 10.1038/s41590-020-00816-x 33257900 PMC8557973

[B30] Whitehead M. J. McCanney G. A. Willison H. J. Barnett S. C. (2019). MyelinJ: an ImageJ macro for high throughput analysis of myelinating cultures. *Bioinformatics* 35 4528–4530. 10.1093/bioinformatics/btz403 31095292 PMC6821319

[B31] Xiao B. Liu L. Li A. Xiang C. Wang P. Li H. (2020). Identification and verification of immune-related gene prognostic signature based on ssGSEA for Osteosarcoma. *Front. Oncol.* 10:607622. 10.3389/fonc.2020.607622 33384961 PMC7771722

[B32] Yang Z. Yang X. Xu S. Jin P. Li X. Wei X. (2017). Reprogramming of stromal fibroblasts by SNAI2 contributes to tumor desmoplasia and ovarian cancer progression. *Mol. Cancer* 16:163. 10.1186/s12943-017-0732-6 29041931 PMC5645935

[B33] Yoshihara K. Shahmoradgoli M. Martínez E. Vegesna R. Kim H. Torres-Garcia W. (2013). Inferring tumour purity and stromal and immune cell admixture from expression data. *Nat. Commun.* 4:2612. 10.1038/ncomms3612 24113773 PMC3826632

[B34] Yu G. Wang L. G. Han Y. He Q. Y. (2012). clusterProfiler: an R package for comparing biological themes among gene clusters. *Omics* 16 284–287. 10.1089/omi.2011.0118 22455463 PMC3339379

[B35] Zhang P. Yang F. Luo Q. Yan D. Sun S. (2019). miR-1284 inhibits the growth and invasion of breast cancer cells by targeting ZIC2. *Oncol. Res.* 27 253–260. 10.3727/096504018x15242763477504 30075825 PMC7848447

[B36] Zhang Y. Du W. Chen Z. Xiang C. (2017). Upregulation of PD-L1 by SPP1 mediates macrophage polarization and facilitates immune escape in lung adenocarcinoma. *Exp. Cell Res.* 359 449–457. 10.1016/j.yexcr.2017.08.028 28830685

[B37] Zhou W. Gross K. M. Kuperwasser C. (2019). Molecular regulation of Snai2 in development and disease. *J. Cell Sci.* 132:jcs235127. 10.1242/jcs.235127 31792043 PMC12233911

[B38] Zhou Z. Liu C. Liu K. Lv M. Li B. Lan Z. (2020). Expression and possible molecular mechanisms of microRNA-205-5p in patients with head and neck squamous cell carcinoma. *Technol. Cancer Res. Treat.* 19:1533033820980110. 10.1177/1533033820980110 33327871 PMC7750893

