# Supplementary figures and images for: Prognostic Immune-Related Genes of Patients With Ewing’s Sarcoma
Source: Front Genet. 2021 May 28;12:669549. doi: 10.3389/fgene.2021.669549 (PMC8194304; doi:10.3389/fgene.2021.669549)

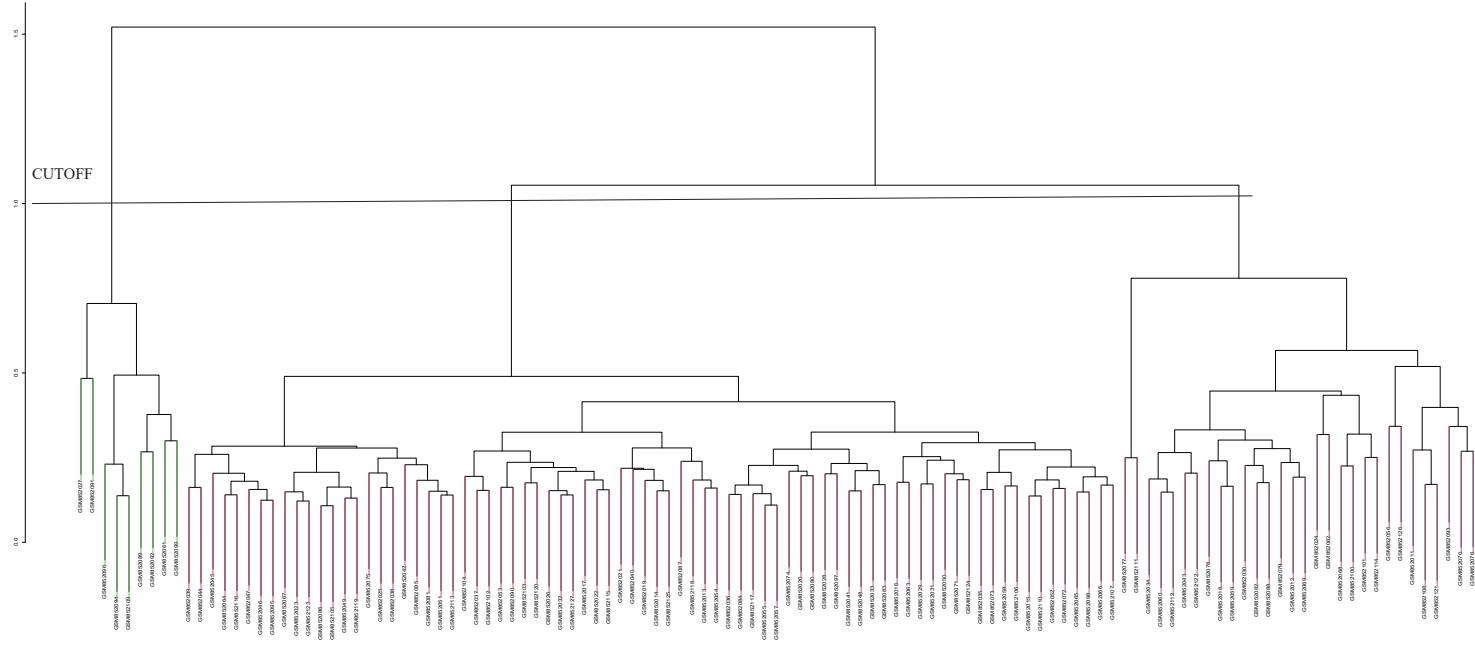

Supplement: Supplementary file 2 [file Data_Sheet_1.PDF]

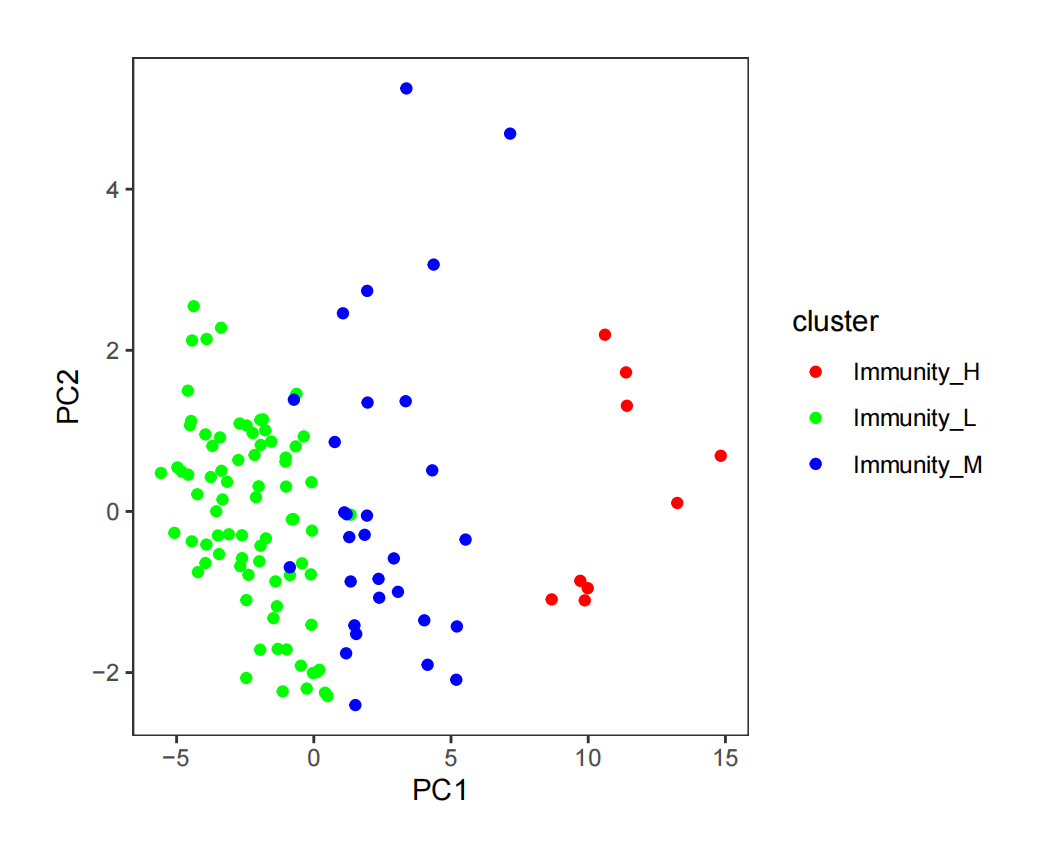

Supplement: Supplementary file 3 [file Image_1.TIFF]
